# Comparing panic alarm systems for high-risk domestic abuse victims: a randomised controlled trial on prevention and criminal justice system outcomes

**DOI:** 10.1007/s11292-022-09505-1

**Published:** 2022-04-04

**Authors:** William Hodgkinson, Barak Ariel, Vincent Harinam

**Affiliations:** 1 Bedfordshire Police, Woburn Rd, Kempston, Bedford, MK43 9AX UK; 2 Institute of Criminology, University of Cambridge, Sidgwick Ave, Cambridge, CB3 9DA UK; 3 Institute of Criminology, The Hebrew University of Jerusalem, Mt. Scopus, 9190501 Jerusalem, Israel

**Keywords:** Police, Domestic abuse, Panic alarms

## Abstract

**Background:**

The use of panic alarm systems for victims of domestic abuse is becoming increasingly popular. However, tests of these devices are rare. Consequently, it is presently unknown whether domestic abuse offenders are deterred by warning stickers informing them that a panic alarm system is installed on the premises, or whether alarm systems reduce domestic abuse recidivism. There is also a lack of data regarding whether adding an audio-recording feature to the panic alarm results in more prosecutions of domestic abuse offenders compared to standard panic alarm systems. Measuring the efficacy of warning stickers and audio recordings will enhance understanding of the overall effectiveness of panic alarm systems for domestic abuse.

**Methods:**

This study used a pre-test-post-test, control group design, in which 300 eligible high-risk domestic abuse victims in London, UK, were randomly allocated to either a standard panic alarm system or a panic alarm system with audio-recording capabilities and a red warning sticker on a durable, A6-size sign displayed at eye level at the entrance to the premises. Each sticker was well lit to ensure maximum visibility. The gain scores of multiple measures at 6 months prior and 6 months post-randomisation were used to assess the treatment effects (including the number of calls for service, recorded crimes, and harm score), and a negative binomial generalised linear model was utilised to estimate the likelihood of criminal charges for domestic abuse offenders in the two systems.

**Outcomes:**

Pre-post comparisons of recidivism suggested an overall reduction in both treatment arms, but there were no statistically significant differences between the two types of alarm systems across these crime measures. Nevertheless, the estimation model indicated a significant 57% increase in charges using the audio-recording alarm relative to the standard panic alarm system.

**Conclusions:**

Using deterrent stickers to warn domestic abuse offenders of panic alarm systems does not lead to a reduction in subsequent harm to victims. Compared to ordinary panic alarms for high-risk domestic abuse victims, audio-recording systems provide valuable evidence that increases subsequent charges, and thus, these systems should be explored further.

**Supplementary Information:**

The online version contains supplementary material available at 10.1007/s11292-022-09505-1.

Reports of domestic abuse (DA) are increasing every year in England and Wales, resulting in 758,941 crime reports between February 2019 and March 2020 (ONS 2020). In the same period, 124 victims of domestic homicide were recorded, giving an average of one homicide every 3 days (ONS 2020). Additionally, the cost of DA was estimated to be £66 billion in 2017 (Oliver et al., 2019). Given the concerning figures associated with DA, it is surprising that there is a lack of high-quality quantitative research focused on police practices for active crime prevention and target hardening.

One method for assisting high-risk victims of DA is through technology such as panic alarms (Blackstone et al., 2020; Hakim et al., 1995). For eligible victims at risk of further victimisation, a system that is directly linked to the police can be installed in their homes. Upon activation, emergency assistance can be provided directly to the victim, thus circumventing the usual call for service. However, the effectiveness of panic alarms in preventing subsequent harm to DA victims is largely unknown.

In this study, we report the results of a randomised controlled trial (RCT) on the effectiveness of a novel panic alarm system compared with the national standard panic alarm system for England and Wales. More specifically, we examine whether an alarm system that is advertised through entry point stickers and includes an audio-recording capability reduces repeat victimisation and harm suffered and improves the criminal justice outcomes when used by high-risk DA victims compared to a system without audio recording or a warning sticker. It is important to note that both types of panic alarms are silent to ensure comparability across the treatment and control groups.

This RCT is set in London, UK, within the jurisdiction of the Metropolitan Police Service (MPS) during 2020. Our design incorporates a simple trickle-flow RCT across 13 MPS boroughs. The identification of suitable cases begins when an officer detects a high-risk DA victim who would benefit from a panic alarm. If the DA victim agrees to the intervention, they would then be randomly assigned to either a national standard alarm or an audio-recording alarm with a visual warning sticker. Therefore, our experiment assesses two types of outcomes, including prevention through the warning sticker and the ability to record crime, as well as evidence-gathering to prosecute in otherwise difficult cases.

## Literature review

### Police prevention of DA

To determine the methods that reduce harm and repeat victimisation in high-risk DA, it is essential to understand the differences between primary, secondary, and tertiary crime prevention. Primary crime prevention is focused on the whole population, whereas secondary crime prevention is more targeted and focuses on those at greater risk. Tertiary crime prevention concentrates on those who have already been victimised (Radford et al., 2011). The police are involved in *prevention* at the secondary and tertiary levels after at least one report has been made to them. Reports to the police of first-time DA involving intimate partners are usually the last instance of DA reported to them (Sherman & Berk, 1984). Indeed, most reported DA, particularly of the high-harm type, occurs without any prior contact with the police and without additional reported incidents (Bland & Ariel, 2015; Bridger et al., 2017).^1^ Furthermore, efforts led by Thornton (2017), Chalkey and Strang (2017), Button et al. (2017), and Barnham et al. (2017) to identify risk factors based on police records concluded that the majority of severe DA cases came ‘out of the blue’ with no prior police awareness about the dyads, hence the difficulty in primary prevention. Overall, it seems that the risk factors with the most robust prediction validity for subsequent, future DA are the offender’s prior suicidal ideation, stalking, and separation from their partner (see Bland & Ariel, 2020; Goussinsky & Yassour-Borochowitz, 2012), but these factors are mostly not found within police records. Therefore, the focus on effective methods for preventing DA harm by the police is on a specific subgroup of DA dyads for whom at least one incident was reported to the police and potential for re-victimisation exists (Sherman et al., 2017).

### Alarms and panic alarms

Personal and property alarms are a widely used tactic aimed at reducing levels of offending or harm. For example, in the USA, approximately 17 million intruder and fire alarms have been installed (Hakim et al., 1995). However, their effectiveness has been questioned, with 94–99% of police responses resulting from false alarms (Blackstone et al., 2020; see also Gaines & Bichler, 2007). Nevertheless, burglar alarms remain ‘the most effective deterring and detecting measure for burglary’ (Blackstone et al., 2020; see also Scott, 2006; however cf. Knutsson, 1984).

In terms of DA prevention, personal panic alarms linked directly to the police have been widely used in the UK, but there is a lack of strong evidence supporting their scope and effectiveness (see more broadly Perkins et al., 2017). The use of panic alarms for DA was first documented by Farrell and Pease (1993) as a basic system that worked with landlines and ‘alarm pendants’, through which automated calls were made to the local police station when the alarm was triggered. However, Walker (2001) examined the effectiveness of panic alarms for preventing repeat victimisation and harm for DA victims and found a minimal deterrent effect on perpetrators, and there have been few rigorous replications of this study (Berry et al., 2014; Breckenridge et al., 2015). One study by Tumen and Ulucan (2019) in Turkey reported an increased likelihood of physical domestic violence against women equipped with panic alarms relative to a synthetic control group, possibly due to a ‘male backlash’ against the empowerment of women from particular socioeconomic backgrounds (Yüksel & Ulucan, 2021). However, we are unaware of any other published studies to this effect.

Moreover, mobile alert systems represent a similar technology to panic alarms. Specifically, the primary difference is that they operate outside of the home and use the global positioning system (GPS) to inform officers of the victim’s location. Investigating this technology in England and Wales, Natarajan (2016) reported case studies in which rapid responses made possible by mobile alert system activations reduced the risk of repeat victimisation. However, to our knowledge, systematic evidence on the effectiveness of these devices under controlled settings is limited.

### Warning notifications

A common preventative tactic against crime is the use of warning stickers to deter offenders from entering the premises. Such low-cost tactics are designed to alert offenders of a heightened risk of apprehension by police (Raphael, 2015). Indeed, these devices are commonly used for protection from domestic or commercial burglary, with varying degrees of success (Kyvsgaard & Sorensen, 2021; Laycock, 1985). In this regard, whilst reductions in burglaries and break-and-enters have been observed, these reductions were limited to the beginning of the study’s observation period (Kyvsgaard & Sorensen, 2021). Additionally, the effect is not ubiquitous across all crime types, such as the consumption of restricted alcohol and drugs (Mackinnon, 1995). We are unaware of previous evaluations of the use of stickers by the police to prevent DA offenders from harming their victims either in their abodes or other areas.

Given the paucity of research in this area of policing, it is useful to examine the informative evidence from other disciplines. Whilst pictorial warnings have been found to effectively increase the intention to quit smoking compared to text warnings alone (Brewer et al., 2016), warning stickers on prescription medicines often fail to attract the attention of the older population (Guy, 2012). An earlier study by Jones and Nowell (1973) identified a link between the colour of visual cues and physiological responses, suggesting that not all warning signs have the same effect. Furthermore, Cohen and Sloan (2016) as well as Nevo et al. (2010) documented that strong visual contrast is needed in visual cues because individuals’ gaze is drawn and attracted to stimulus changes. Therefore, whilst stickers have been widely used to deter unwanted behaviours, evidence exists to both support and oppose the hypothesis that warnings ubiquitously affect choices to comply with certain rules.

## Methods

### Design

We designed an experiment to assess the effectiveness of a panic alarm system provided by the police to DA victims who are at risk of further victimisation. We could not test the effect of the absence of the system, since a no-treatment condition would involve placing victims deemed as high risk of further victimisation by the police at even greater risk. Therefore, we do not have counterfactual conditions, as it would be unethical to deprive DA victims of the immediate protection provided by panic alarms. Conversely, this study investigates the relative effect of a novel system incorporating both a warning sticker outside the victim’s premises and audio-recording capabilities compared with the existing ‘call for immediate assistance-only’ panic alarms. We estimate the relative effects of each system concurrently, despite the risks to the internal validity of using simple before-after designs such as this (see Cook et al., 2002).

### Setting

The setting for this research was London and the MPS area. The population of London was 8,904,081 in 2018 (London Councils, 2018), and this research was conducted within 13 of the 32 local authority areas and within five of the MPS’s 12 Basic Command Unit (BCU) areas. The trial area contained 3,676,154 people, accounting for 41.29% of London’s population (London Councils, 2018). This RCT commenced on 20 February 2020 and continued until 27 May 2020 when case 300 was achieved. Analysis of DA victimisation focused on the 6-month period prior to and following panic alarm request. The study ended on 27 November 2020. It is important to note that the pre-test took place during the period prior to the COVID-19 pandemic, whereas the post-test took place after the pandemic, which has direct implications for the external and internal validity of the study, especially the pre-post only comparisons. During this period, there were lockdowns in the UK that confined many individuals to their houses, thus changing not only crime but also police response to crime, including domestic abuse (see Nivette et al., 2021; Walklate et al., 2022).

### Eligibility

Police officers operating across geographical areas utilised professional judgement (as opposed to risk assessment frameworks such as Domestic Abuse, Stalking, and Harassment [DASH] or the Domestic Abuse Risk Assessment [DARA]) to identify eligible cases of high-risk DA. Professional judgement about installing panic alarms for high-risk victims is standard practice within the organisation that undertook this experiment, as officers assess whether a panic alarm may assist in managing the risk to the victim. When it was determined that an alarm would mitigate or reduce the threat, the officer contacted the victim to ascertain whether they would agree to an alarm being installed. Upon agreement, the officer applied for an alarm for the victim.

### Treatments

After receiving an alarm request, the case was randomised to receive either an audible alarm (model RDA3) or a standard alarm (model RDA2). Following this, an engineer was instructed to install either an RDA2 or an RDA3 within 24 h of random allocation. Additionally, those who received an RDA3 also received an RDA2 as a backup in the event that the RDA3 failed to operate.

The alarm systems are a handheld ‘fob’ with two buttons, and both buttons had to be pressed simultaneously for 2 s to activate the alarm. Activation of the audio alarm generated the same immediate dispatch of a police unit to the victim’s abode as the standard alarm. However, for the audio alarm, the call type detailed on the message was ‘Audible Panic Alarm Activation’ as opposed to ‘Panic Alarm’. Upon activation in an emergency situation (i.e., when the suspect appeared at the home of the victim), operatives in a control room received an alert with pre-recorded details of the location, victim, and case type, and a Computer-Aided Dispatch (CAD) reference was then generated. The CAD was passed to a dispatcher operating within the defined BCU area, and the dispatcher communicated the need for an immediate response to the pre-assigned location due to a high-risk DA alarm activation. A police unit was then dispatched to the location on an immediate grade (I Grade, which has a 15-min response time attached) call, and the responding officers were directed to turn on their body-worn video (BWV) cameras. The process of dispatching officers to the victim was similar in both experimental arms, except officers responding to addresses with an RDA3 were also informed that an audio recording would be available, and they were required to review the recording at the scene.

#### Unique features of the RDA3 system

The primary difference between the two systems is that the RDA3 also records audio within the household following activation. Furthermore, the RDA3 technology was accompanied by visual warning stickers, which informed people entering the property that audio-recording technology was present at the address. The signage was large enough to be noticeable during both day and night, was positioned at eye level on the front door of the victim’s home (usually apartment), and was bright red in colour to attract attention. Therefore, the RDA3 treatment arm possessed five key elements that were not present in the RDA2 treatment arm:

1. The capturing of audio recording within the address from the time of alarm activation and 15 minutes of audio prior to activation.
2. The ongoing recording and review until police arrival.
3. The notification to police that audio recording was taking place and would be available for review.
4. The presence of deterrent stickers at points of access to the property.
5. Governance to ensure that investigating officers were aware of the presence of audio evidence.

### Randomisation

We used a trickle-flow randomisation sequence. Once a DA case was deemed eligible, the individual would be randomly assigned using an automated allocation generator (Linton & Ariel, 2020). No baseline covariates were used to create balance, and the randomisation ‘forced’ a 50:50 split between treatment and control conditions. Intention-to-treat approach was used in this RCT but, overall, 75% of victims accepted the alarm system assigned to them.

### Data sources

We used two police data-recording systems: the crime reporting information system (CRIS) and MPS’s call management system (CMS). Serving as MPS’s data management system, CRIS captures data on the victim, prior offences against them, the arrest status of named suspects, the type of caller, and outcomes of the investigations. CRIS data are often inaccurate as the same victim can be inputted with slightly different name spellings. Indeed, unlike countries such as the USA, the UK does not have a system to identify individuals using a unique ID number. This issue meant that accurate data could not be easily retrieved from a simple search of the CRIS. To overcome this challenge, searches were completed using the victim’s forename, surname, date of birth, and address, as well as the same corresponding categories for the offender. The data search parameters covered offences against the victim in the 6 months prior to and the 6 months after the alarm request.

The second data source, CMS, contained information on the alarm unit reference numbers, date of request, date of installation, sticker placement, any alarm installation refusals, and the reason for refusals. CMS was the system used before police dispatch via CAD. CMS captures all call demands to the MPS and categorises demand based on caller type.

### Measures

#### Calls for service

‘999’ emergency calls for service for DA, made either by the victim or a third party, were used as the first of three primary outcomes. For each case, we measured the number of emergency calls 6 months pre- and 6 months post-random assignment.

#### Crimes

Once a call for service incident is ‘crimed’ by a police officer, it is registered as a crime on CRIS. Not all calls for service are crimed due to misclassification or a lack of evidence. CRIS data are considered more precise than calls for service, as more investigatory efforts are applied to the incident. However, these data were filtered using search terms in the database and, as a result, susceptible to human errors. We observed both DA crimes and all other crimes at 6 months pre- and post-random assignment. It should be mentioned that a thorough investigation was conducted for every call for service and, following this, a flagged DA was applied to the CRIS database accordingly.

#### Harm

There are different approaches to estimating the harm caused by a given crime (see recent review in Qureshi et al. (2021)). One recent approach involves employing the Cambridge Crime Harm Index (CCHI), which uses the England and Wales sentencing guidelines to attribute harm to crimes based upon the number of prison days; higher values on the CCHI indicate that more harm is assumed to be caused by that crime category (see Sherman et al. (2017) for further details). Crime harm measures such as the CCHI present objective measurements of crime harm that are unaffected by the biases of victim reporting and sentences meted out by judges. Using this measure of crime harm at 6 months pre- and post-intervention allowed us to observe variations in harm scores.

#### Charges

The count of charges was measured post-random assignment only. We could not compute pre-test scores because many cases would have continued over the treatment period, as the decision to pass the case to the Crown Prosecution Services can take a long time. Higher levels of this output variable are interpreted as a positive change, as the treatment arm in which a charge is recorded means the case had sufficient evidence to press charges. It is important to mention that charges are recorded on the crime report system CRIS and are not a court outcome; in the UK context, charges represent the decision that there is a probable but not definite prospect of conviction.

#### Alarm activations

The number of activations post-randomisation was counted in each treatment arm to determine the extent of the use of the devices by victims.

### Statistical analyses

We used a series of tests to estimate the treatment effect. We first determined descriptive statistics and before-after only comparisons to illustrate the overall patterns in the data. Following this, we used the gain score approach for analysing the pre-test-post-test control group design (Campbell & Stanley, 1963, pp. 23–24). For each study group, we computed the pre-test-post-test gain scores and then conducted independent sample *t*-tests between the two treatment arms on these gain scores. The scores were normally distributed (see Supplementary Materials A). We supplemented these analyses with Cohen’s *d* and the associated 95% confidence intervals (CI) to estimate the magnitude of the differences between the two treatment groups.

Furthermore, we modelled the likelihood of charging by predicting the number of charges in each arm based on group and including the activation of the alarm device as a covariate. Given the count distribution of the outcome variable and the over-dispersed nature of charges (i.e., mostly nil counts), we used a negative binomial generalised linear model with robust estimators. We have provided the 95% CI for the Wald statistic, as well as the respective *p*-values for each parameter.

## Findings

**Fig. 1:**
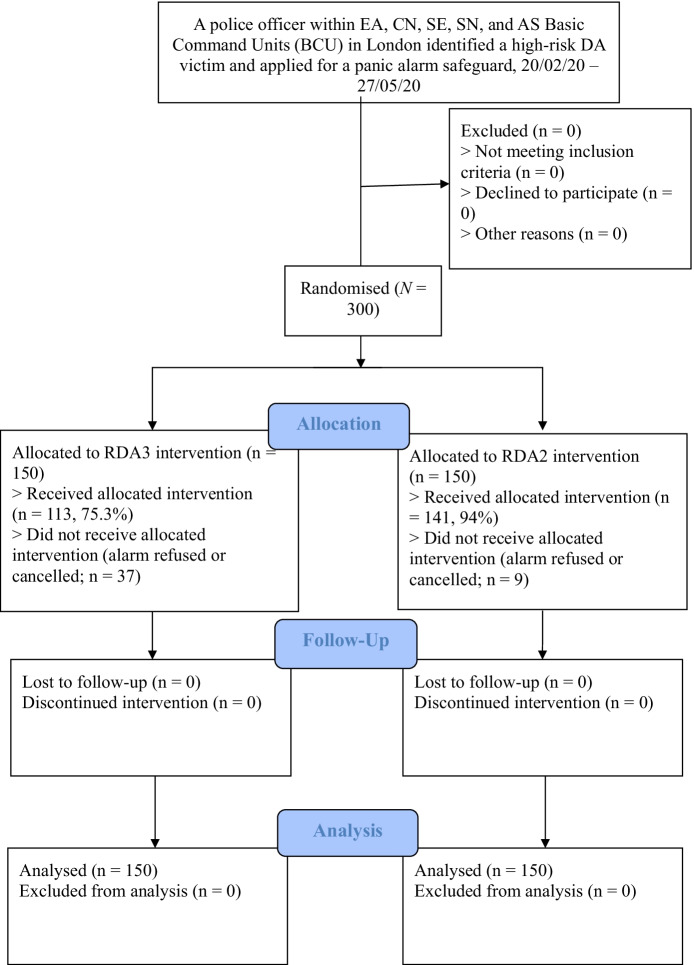
CONSORT flowchart

In total, 300 alarms were requested and randomised. An overall fidelity rate of 84.7% was achieved, corresponding to 254 individuals receiving the treatment out of 300 cases. In total, 141 (94%) and 113 (75.3%) victims received an RDA2 and RDA3, respectively. The CONSORT flowchart is presented in Fig. 1.

### Pre-test measures

 Considering the baseline data on the 300 victims (see Table 1), a total of 1247 calls for service were made to the police (*M* = 4.16), with a total of 1559 police units deployed to these calls, and there were no statistically significant differences between the two arms (*t*(298) = 0.53, *p* = 0.60). We also observed 648 crimes recorded in the 6 months prior to randomisation, of which 578 were flagged as DA-related. These figures highlight the gap that emerges between calls made to the police and crimes recorded by the police as DA (more than half of calls for service are not converted into crimes). There were no observable differences between the treatment groups at baseline in terms of all crimes (*t*(298) =  − 0.27; *p* = 0.79) or DA-flagged crimes (*t*(298) =  − 0.23, *p* = 0.82). The total CCHI score for the pre-trial period was 397.45 on average but with a large variability (SD = 819.01), although there were similar distributions between the two study arms (*t*(298) = 0.62, *p* = 0.53 for all crimes; (*t*(298) = 0.56, *p* = 0.58) for DA crime harm only.

**Table 1:** Pre-test measures

Outcome	All crime reports			DA-only crime reports		
	RDA2	RDA3	Total	RDA2	RDA3	Total
*Crime count*						
0	7	9	16	10	11	21
1	66	55	121	71	62	133
2	36	42	78	36	37	73
3	18	18	36	16	21	37
4	8	11	19	5	11	16
5	4	7	11	3	4	7
6	5	4	9	4	2	6
7	3	2	5	4	1	5
8	2	1	3	1	0	1
9	1	0	1	0	0	0
10	0	1	1	0	10	1
Total crime count	320	328	648	286	292	578
Mean	2.13	2.19	2.16	1.91	1.95	1.93
Standard deviation	1.74	1.69	1.71	1.58	1.48	1.53
Range	0–9	0–10	0–10	0–8	0–10	0–10
*Crime harm*						
Total crime harm (CCHI)	64,042.75	55,190.75	119,233.5	62,828.25	54,964.5	117,792.75
Mean per group	426.95	367.94	397.45	418.86	366.43	392.64
Standard deviation	901.23	729.45	819.01	898.05	729.24	817.06
Range	0–4757	0–3297	0–4757	0–4755	0–3297	0–4755
*Calls for service*						
Total calls for service	599	648	1247	-	-	-
Mean per group	3.99	4.32	4.16	-	-	-
Standard deviation	5.61	5.13	5.37	-	-	-
Range	0–30	0–32	0–32	-	-	-
*Police units deployed*						
Total police units deployed	702	857	1,559	-	-	-
Mean per group	4.68	5.71	5.20	-	-	-
Standard deviation	1.49	1.78	1.65	-	-	-
Range	0–14	0–20	0–20	-	-	-

### Pre-post comparisons

As shown in Table 2, the total crime count for the 6-month post-intervention period was 336, split evenly between treatment groups. We note that there were 312 fewer counted crimes in the post-intervention period compared to the pre-intervention period, corresponding to − 1.04 crimes per victim. Whereas victims in the RDA2 treatment group had a before-after reduction of 47.5% (152; − 1.01 reports per victim), those in RDA3 had a before-after reduction of 48.8% (160; − 1.07 reports per victim). Overall, the pre-test-post-test reductions were statistically significant for both treatment groups (*t*(149) =  − 6.4, *p* < 0.001).

**Table 2:** Post-test outcome measures

Measurements	All crime reports			DA-only crime reports		
	RDA2	RDA3	Total	RDA2	RDA3	Total
*Crime count*						
0	80	76	156	90	87	177
1	32	34	66	31	28	59
2	19	20	39	15	17	32
3	7	6	13	6	6	12
4	3	4	7	1	7	8
5	5	4	9	4	2	6
6	0	3	3	1	1	3
7	1	2	3	0	1	1
8	1	1	2	0	0	0
9	0	0	0	0	0	0
10	0	0	0	0	0	0
11	1	0	1	1	0	1
12	0	0	0	0	0	0
13	0	0	0	1	0	1
14	1	0	1	0	0	0
Total crime count	168	168	336	133	137	270
Mean per group	1.12	1.12	1.12	0.89	0.91	0.90
Standard deviation	1.98	1.67	1.83	1.77	1.49	1.62
Range	0–14	0–8	0–14	0–13	0–7	0–13
*Crime harm*						
Total crime harm	9549.5	13,312.25	22,861.75	7211.5	12,700.05	19,911.55
Mean per group	63.66	88.75	76.21	48.08	85.24	66.60
Standard deviation	474.46	370.75	425.25	336.74	500.82	358.21
Range	0–5505.75	0–3285	0–5505.75	0–3680.8	0–3285	0–3680.8
*Calls for service*						
Total calls for service	620	699	1319	-	-	-
Mean per group	4.13	4.66	4.40	-	-	-
Standard deviation	6.07	6.37	6.22	-	-	-
Range	0–50	0–40	0–50	-	-	-
*Police units deployed*						
Total units deployed	748	851	1599	-	-	-
Mean per group	4.99	5.67	5.33	-	-	-
Standard deviation	1.6	1.77	1.69	-	-	-
Range	0–16	0–16	0–16	-	-	-

A similar pre-test-post-test pattern was observed for DA-only crime reports in both treatment arms. There was a total before-after reduction of 308 DA crime reports (− 1.03 per victim). Furthermore, those in the RDA2 group had a reduction of 46.5% (153; − 1.02 reports per victim), whilst the reduction for DA crime reports in the RDA3 group was 46.9% (155; − 1.03 reports per victim). These results demonstrate that both the RDA2 (*t*(149) =  − 7, *p* < 0.001) and RDA3 (*t*(149) =  − 7.4, *p* < 0.001) groups showed statistically significant reductions in DA crime reports.

The total crime harm for the post-intervention period was 96,371.75, giving an overall reduction in crime harm of 80.8% or − 321.2 per victim. Whereas victims in the RDA2 treatment group had a before-after harm reduction of 85.1% (− 54,493.25; − 362.34 reports per victim), those in the RDA3 group had a before-after reduction of 75.9% (− 41,878.5; − 279.2 reports per victim). The RDA2 (*t*(149) =  − 5.1, *p* < 0.001) and RDA3 (*t*(149) =  − 4.1, *p* < 0.001) groups both demonstrated statistically significant reductions in crime harm. With regard to DA, there was a total before-after reduction of 83.1% (− 97,881.2; − 326.04 per victim). Those in the RDA2 group had an 88.5% reduction (55,616.72; − 370.78 reports per victim), whilst those in the RDA3 group had a 76.9% reduction in crime harm (− 42,264.5; − 281.19 reports per victim). Overall, the RDA2 (*t*(149) =  − 5.32, *p* < 0.001) and RDA3 (*t*(149) =  − 4.11, *p* < 0.001) groups both showed statistically significant reductions in crime harm.

Finally, Table 3 presents the calls for service by caller type. The number of victim callers reduced by 37.2% and 42.6% for RDA2 and RDA3, respectively. Concurrently, the number of other caller types in both groups increased from the pre-intervention to the post-intervention period. The number of calls increased by 51 in the RDA3 treatment group, giving a mean increase of + 0.34 per victim, whilst the number of calls increased by 21 CFS in the RDA2 treatment group, giving a mean increase of + 0.14 per victim. Neither of these increases were statistically significant (RDA2: *t*(149) = 0.33, *p* = 0.74; RDA3: *t*(149) = 0.72, *p* = 0.47). With regard to police units deployed, a slight mean reduction was observed in the RDA3 group (− 0.04 per victim), whilst there was a small increase for the RDA2 group (0.31 per victim). Neither of these changes between the pre-intervention and post-intervention periods were statistically significant (RDA2: *t*(149) = 0.6, *p* = 0.56; RDA3: *t*(149) =  − 0.06, *p* = 0.95).

**Table 3:** Calls for service by caller type

	Pre-test		Post-test	
Caller type	RDA2	RDA3	RDA2	RDA3
Other	33 (5.51%)	119 (19.19%)	12 (1.85%)	130 (18.6%)
Staff on duty	59 (9.85%)	75 (12.1%)	87 (13.43%)	87 (12.45%)
Third party	266 (44.41%)	265 (42.74%)	281 (43.36%)	318 (45.49%)
Unlisted/unknown	13 (2.17%)	5 (0.81%)	16 (2.47%)	16 (2.29%)
Victim	207 (34.56%)	132 (21.29%)	230 (35.49%)	132 (18.88%)
Witness	21 (3.51%)	24 (3.87%)	22 (3.40%)	16 (2.29%)
**Total**	**599 (100%)**	**620 (100%)**	**648 (100%)**	**699 (100%)**

Bolded figures simply reflect total numbers. They are an aesthetic feature within the table more than anything else

### Between-group comparisons

Table 4 summarises the results of our statistical tests on the treatment effect, including the between-group pre-test-post-test gain scores, *d*, and the corresponding 95% CI. As shown, there were no statistically significant differences between the two groups across all crime measures. Considering the before-after comparisons reported above, the between-group variations were similar and did not indicate significant gains for one system compared to the other in terms of calls for service to the police, total crimes, DA crimes, CHI, or CHI for DA.

**Table 4:** Pre-test-post-test between-group gain scores: total crimes, DA crimes, harm, DA harm, and calls for service

		*N*	Mean	Std. deviation	Std. error mean	*t*	*p*	*d*	95% CI	
Crimes	RDA2	150	− 1.01	1.942	0.159	0.231	0.817	0.027	− 0.200	0.253
	RDA3	150	− 1.07	2.049	0.167					
DA crime	RDA2	150	− 1.02	1.774	0.145	0.100	0.920	0.012	− 0.215	0.238
	RDA3	149	− 1.04	1.716	0.141					
CCHI	RDA2	150	− 363.29	872.762	71.261	− 0.855	0.393	− 0.099	− 0.325	0.128
	RDA3	150	− 279.19	829.402	67.720					
DA CCHI	RDA2	150	− 370.78	854.330	69.756	− 0.888	0.375	− 0.103	− 0.330	0.124
	RDA3	149	− 283.66	841.137	68.909					
Calls for service	RDA2	150	0.14	5.185	0.423	− 0.315	0.753	− 0.036	− 0.263	0.190
	RDA3	150	0.34	5.796	0.473					

*CCHI* Cambridge Crime Harm Index, *DA* domestic abuse, *CI* confidence interval, *RDA2* non-recording alarm, *RDA3* audio-recording alarm

From a substantive perspective, it is clear that the magnitude of the differences between the two groups is minimal across all crime outcome measures, with marginal to small effect sizes throughout that are predominantly located near the null line (Cohen, 1980). Indeed, this suggests that neither treatment is preferable for reducing repeat calls for service to the police, recorded crimes, and harm scores.

### Modelling likelihood of charges based on RDA2 and RDA3 activations

In total, the trial generated 153 alarm activations from 77 discrete victims out of a total of 300 participants. RDA3 victims activated their alarms 93 times, whilst those in the RDA2 group made 60 activations. Importantly, alarm activations in the post-intervention period resulted in 28 charges, with 10 and 18 in the RDA2 and RDA3 groups, respectively.

Our statistical model (Table 5) suggests a significant effect of RDA3 treatment compared to RDA2, with substantial gains in charges (*B* =  − 1.377, standard error [SE] = 0.652, *p* = 0.035), as well as statistically significant interaction term between treatment condition and system activation, with those in the RDA3 group being more likely to activate their alarm and to result in a charge (*B* = 0.793, SE = 0.34, *p* = 0.021). The estimated marginal means of these outcomes suggest 0.03 charges (SE = 0.01) for RDA2 versus 0.07 charges (SE = 0.02) for RDA3, or a 57% change between the two systems.

**Table 5:** Parameter estimates for charges post-randomisation (RDA2 vs RDA3) — negative binomial regression results

Parameter	*B*	Std. error	95% Wald CI		*p*
			Lower	Upper	
*Intercept*	− 2.925	0.4274	− 3.762	− 2.087	0.000
(Random assignment = RDA2)	− 1.377	0.6524	− 2.656	− 0.099	0.035
Total alarm activations post-randomisation	0.528	0.2764	− 0.013	1.070	0.056
(RDA2 * total alarm activations post-randomisation)	0.793	0.3432	0.120	1.465	0.021

Model: (intercept), random assignment, total alarm activation post-randomisation, random assignment * total alarm activation post-randomisation

## Discussion

This study aimed to examine the effects of two types of panic alarm systems on a number of outcomes for DA victims. Overall, the between-group comparisons in this study suggest no substantive or statistically significant treatment effects on crime counts, DA crime counts, crime harm (CCHI), DA harm (CCHI), calls for service, or the overall number of police units deployed. With a good sample size and relatively sufficient follow-up for each unit, our study suggests that neither system of panic alarms for DA victims produces greater prevention effects. RDA3 is an alarm system that aims to deter DA offenders via a visible sticker at the entrance to the premises warning them that an audio-recording system is present, but this system does not seem to generate a significant deterrent effect and, thus, provides no apparent benefit in terms of prevention. In contrast to our hypothesised effect, notifying DA offenders with stickers about the presence of a panic alarm system does not result in further reduction of harm to DA victims. Indeed, the very least we can conclude is that neither system is worse relative to the other.

At the same time, our model suggests a relatively strong and clinically significant effect of RDA3 in terms of providing evidence that can then be used in criminal charges against DA offenders. Relative to RDA2, which does not have recording capabilities, RDA3 produced a 57% increase in charges. We interpret this finding as carrying crucial practical significance, especially given the low rates of prosecution for DA. In this regard, the audio evidence assisted police in providing additional evidence linking the offender to the scene based on voice recognition. This incriminating evidence is commonly unavailable in DA crimes, which makes prosecuting offenders very difficult (Petersen et al., 2021), Therefore, if evidence captured at the scene can be used to bring DA offenders to justice, the implications are far-reaching, despite the fact that subsequent harm was not prevented above and beyond the effects of the non-recording RDA2 system.

Although before-after designs are weaker than others in terms of methodological rigour, the findings suggest strong before-after effects of both alarm systems, with significant reductions in subsequent reporting and recording of crime, as well as harm to DA victims. A more conclusive finding regarding the efficacy of panic alarms requires the estimation of treatment effects compared with no-treatment groups, which were not feasible in the present experiment. Indeed, other factors may explain the substantial reductions observed in the before-after comparisons (e.g., incapacitation of the offenders, deterrence of the offenders by police actions, self-protection by the victims, or greater care by the police). However, if some of the reduction in harm to victims can be attributed to the panic alarms, then we recommend testing the use of these systems with more victims than those presently given these devices. Overall, further experiments, preferably with more diverse populations of DA types, are therefore warranted.

### Additional limitations

Taken together, our evidence suggests that there is an overall reduction in DA once any panic alarm system is introduced. At the same time, the substantial reductions across all measures could be explained by any number of covariates, including differences in crime patterns during the COVID-19 period (see Nivette et al., 2021); police deterrence (see Sherman, 1998); termination of the relationship; capacity for independence within a wider context of sociodemographic, economic, and cultural factors; and other legal instruments such as protection orders (Dowling et al., 2018). As a result, we cannot robustly conclude an overall treatment effect with this pre-post-only design (see review in Sect. 4 in Ariel et al. (2022)), and more sophisticated methodologies are needed. However, if our causal estimates are valid, then panic alarms of any kind may represent a useful instrument in the prevention of subsequent harm to DA victims who are deemed as high-risk cases by the police.

Taking our before-after findings at face value, our study contradicts Tumen and Ulucan’s (2019) study, which found an increased likelihood of physical domestic violence against Turkish women equipped with panic alarms. Differences may be attributed to contextual differences in households and families between the UK and Turkey, differences in experimental designs (synthetic control group versus before-after only comparisons), the type of technology used, and cultural differences between the participants. These inconsistencies call for more research in this sphere, given the mixed results of the two studies.

Furthermore, we had no access to the audio recordings from the RDA3 panic alarms, and, thus, cannot ascertain how likely the captured evidence was to vindicate or incriminate those accused of DA. Given the overall context, whereby the police and its partner agencies deem cases as having a high risk of recidivism, often of the most violent or controlling type, then we can assume that the evidence often sides with the victim. Unlike the more common high-frequency, low-harm DA that the police often have to deal with (Bland & Ariel, 2020), victims equipped with panic alarms are at the ‘high end’ of both harm as well as risk. More research is needed as to the nature of the evidence captured by the audio system, but we nevertheless hold a strong assumption that the evidence is incriminating rather than vindicating.

Finally, we conclude that the evidence that would lead to charges and possible incarceration is critical, and an alarm system with audio-recording capabilities can be helpful. However, whether additional arrests and, possibly, further prosecution achieve a reduction in subsequent harm remains contestable (see Sherman & Harris, 2015a, b), and we are not able to report the distal effects on court outcomes. However, these audio recordings at least pave the way towards potentially reducing harm through the criminal justice system. Future research should incorporate these outcomes in assessing the technological approaches used by the police.

## Supplementary Information

Below is the link to the electronic supplementary material.

Supplementary file1 (DOCX 217 KB)

## Footnotes

1 However, we note that such conclusions are based on official crime data rather than self-reported incidents, so ‘first-time’ and ‘last-time’ definitions are unlikely to provide information about the entire abuse cycle. Differentiating between the two data sources is important, as much of domestic abuse goes unreported.
