# Supplementary material for: Comparing panic alarm systems for high-risk domestic abuse victims: a randomised controlled trial on prevention and criminal justice system outcomes
Source: J Exp Criminol. 2022 Apr 4:1–19. Online ahead of print. doi: 10.1007/s11292-022-09505-1 (PMC8979151; doi:10.1007/s11292-022-09505-1)
Supplement: Supplementary file 1 — Supplementary file1 (DOCX 217 KB) [file 11292_2022_9505_MOESM1_ESM.docx]

**Supplementary Materials A**

Crime, DA Crime, CCHI, and CCHI Harm Gain Scores

GGRAPH

/GRAPHDATASET NAME="graphdataset"

VARIABLES=Diff_Harm[LEVEL=scale] Diff_DVHarm[LEVEL=scale] Diff_DV_Crime[LEVEL=scale]

Diff_CFS[LEVEL=scale] Diff_Crime[LEVEL=scale]

MISSING=LISTWISE REPORTMISSING=NO

/GRAPHSPEC SOURCE=VIZTEMPLATE(NAME="Scatterplot Matrix (SPLOM)"[LOCATION=LOCAL]

MAPPING( "all"="Diff_Crime"[DATASET="graphdataset"]

"all"="Diff_DV_Crime"[DATASET="graphdataset"] "all"="Diff_Harm"[DATASET="graphdataset"]

"all"="Diff_DVHarm"[DATASET="graphdataset"] "all"="Diff_CFS"[DATASET="graphdataset"]))

VIZSTYLESHEET="Traditional"[LOCATION=LOCAL]

LABEL='SCATTERPLOT MATRIX (SPLOM): Diff_CFS-Diff_Crime-Diff_Harm-Diff_DVHarm-Diff_DV_Crime’

DEFAULTTEMPLATE=NO.


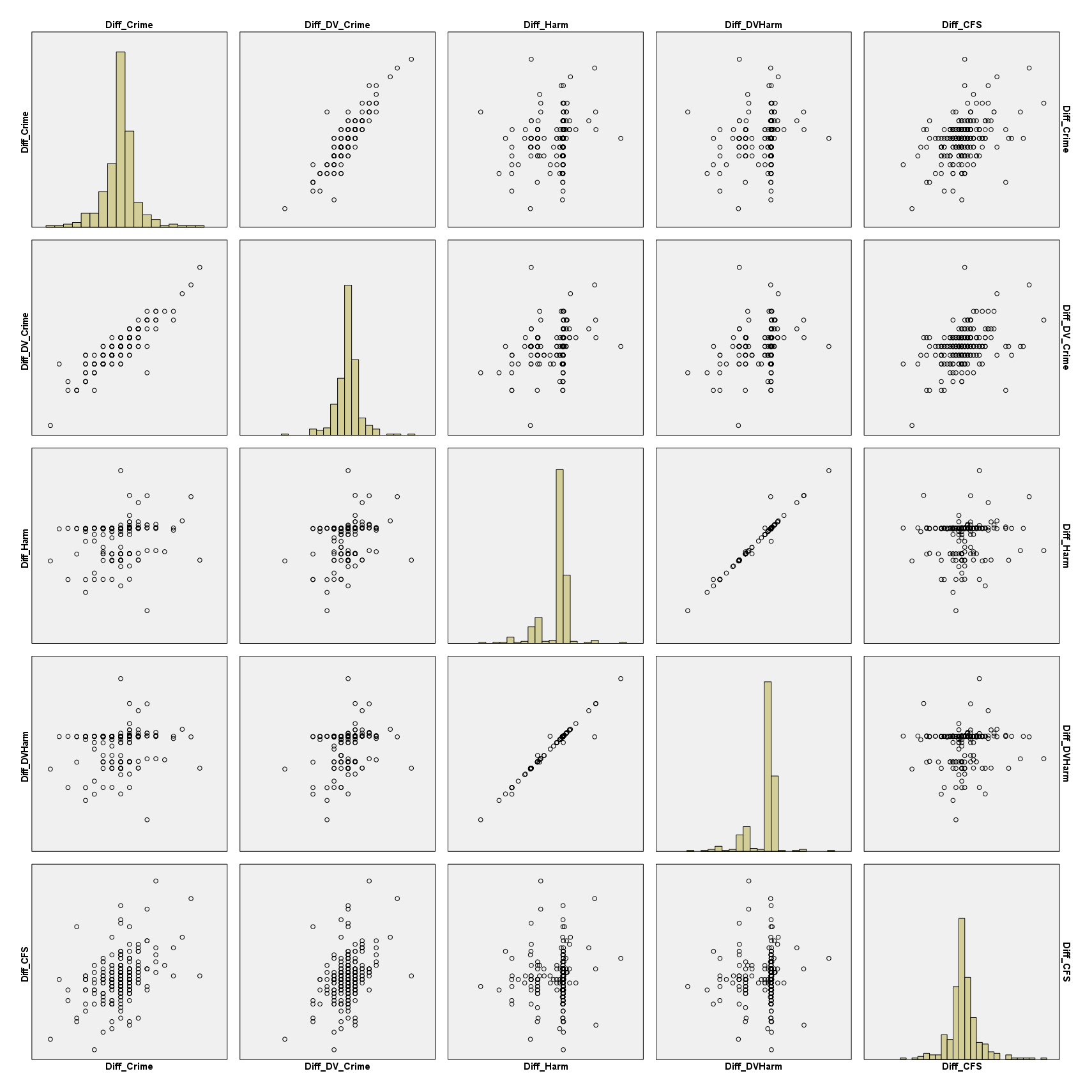


Dif_Crime = post-test-pre-test gain scores for crimes recorded by the police (six months before and after randomisation)

Diff_DV_Crime = post-test-pre-test gain scores for domestic abuse (DA) crimes recorded by the police (six months before and after randomisation)

Dif_Harm = post-test-pre-test gain scores for Cambridge Crime Harm Index Scores (CCHI) six months before and after randomisation

Diff_DV_HARM = post-test-pre-test gain scores for DA CCHI (six months before and after randomisation)
